# Clinical integration of fast Raman spectroscopy for Mohs micrographic surgery of basal cell carcinoma

**DOI:** 10.1364/BOE.417896

**Published:** 2021-03-11

**Authors:** Radu Boitor, Coen de Wolf, Frank Weesie, Dustin W. Shipp, Sandeep Varma, David Veitch, Aaron Wernham, Alexey Koloydenko, Gerwin Puppels, Tamar Nijsten, Hywel C. Williams, Peter Caspers, Ioan Notingher

**Affiliations:** 1 School of Physics and Astronomy, University of Nottingham, University Park, Nottingham, NG7 2RD, United Kingdom; 2 Department of Dermatology, Erasmus MC, Rotterdam 3015 GD, The Netherlands; 3 Nottingham NHS Treatment Centre, Nottingham University Hospitals, Lister Rd, Nottingham NG7 2FT, United Kingdom; 4 Mathematics Department, Royal Holloway University of London, Egham, TW20 OEX, United Kingdom; 5 Center for Optical Diagnostics and Therapy, Department of Dermatology, Erasmus MC, Rotterdam 3015 GD, The Netherlands; 6 RiverD International B.V., Marconistraat 16, Rotterdam 3029 AK, The Netherlands; 7 Centre for Evidence Based Dermatology, Nottingham University Hospital NHS Trust, QMC Campus, Derby Road, Nottingham NG7 2UH, United Kingdom

## Abstract

We present the first clinical integration of a prototype device based on integrated auto-fluorescence imaging and Raman spectroscopy (Fast Raman device) for intra-operative assessment of surgical margins during Mohs micrographic surgery of basal cell carcinoma (BCC). Fresh skin specimens from 112 patients were used to optimise the tissue pre-processing and the Fast Raman algorithms to enable an analysis of complete Mohs layers within 30 minutes. The optimisation allowed >95% of the resection surface area to be investigated (including the deep and epidermal margins). The Fast Raman device was then used to analyse skin layers excised from the most relevant anatomical sites (nose, temple, eyelid, cheek, forehead, eyebrow and lip) and to detect the three main types of BCC (nodular, superficial and infiltrative). These results suggest that the Fast Raman technique is a promising tool to provide an objective diagnosis “tumour clear yes/no” during Mohs surgery of BCC. This clinical integration study is a key step towards a larger scale diagnosis test accuracy study to reliably determine the sensitivity and specificity in a clinical setting.

## 1. Introduction

Basal cell carcinoma (BCC) is the most common type of cancer in humans, with ∼5.7 million cases diagnosed worldwide [1]. Mohs micrographic surgery is used to treat large BCCs (>2cm), lesions occurring on the head-and-neck areas, as well as recurrent BCCs. Research has shown that Mohs surgery provides lower tumour recurrence and provides better conservation of healthy tissue compared to wide-local excision of BCC [2,3]. In Mohs surgery, sequential thin layers of skin are removed and checked by frozen section histology to detect residual tumour [4]. While effective at removing the tumour with minimal damage to healthy tissue, Mohs surgery is expensive due to its reliance on histopathology and availability of specialist surgeons trained to interpret histopathology sections [5].

The efficacy of Mohs surgery may increase if the surgical margins could be analysed faster, more reliably and without requiring tissue processing (freezing, cutting and staining) While multiple techniques were employed for the investigation of BCC *in-situ*, few have been utilised *ex-vivo*, to investigate the resection surface of skin specimens excised in Mohs surgery [6–8]. Fluorescence confocal microscopy (FCM) scans the tissue surface with a laser beam that is selected to excite fluorophores that bind to cell nuclei [7,9,10]. It produces images of the tissue that highlight nuclei which can be used by trained clinical staff to identify BCC. Earlier work indicated a sensitivity and specificity of 88% and 99%, respectively [7], and a more recent diagnostic test of accuracy study (753 fresh Mohs specimens) reported a sensitivity of 79.8% and a specificity of 95.8%, when compared with frozen section histopathology [11]. Recent studies have shown that FCM images can be digitally coloured to appear similar to H&E sections [12]. Peters *et al.* reported that BCC was identified with a 73% sensitivity and a 96% specificity in digitally coloured FCM images of 544 fresh specimens from 148 BCC excisions [13].

Raman spectroscopy is based on inelastic scattering of light by molecules in tissue and was shown to detect endogenous molecular differences between BCC and healthy skin [14,15]. While Raman mapping is too slow for intra-operative analysis of tissue, we developed a faster multi-modal approach that combines auto-fluorescence confocal microscopy and Raman spectroscopy [16,17]. First, an auto-fluorescence image of the tissue is recorded (405 nm excitation) to obtain the morphology of the tissue and identify collagen-rich normal skin tissue (high fluorescence intensity). An algorithm then divides the remaining area of the image into segments based on fluorescence intensity, from which then Raman spectra are measured. The Raman spectra are then analysed using multivariate spectral classification models in order to establish whether residual BCC is found or not at the surface of the tissue.

Recently, we reported the development of a table-top prototype device based on this technique (Fast Raman device), which indicated promising results in a laboratory setting, when investigating frozen skin specimens [18]. The results showed that the instrument produced repeatable results and could be operated by clinical users after only a few hours of training.

In this study, we integrated the Fast Raman device into the Mohs clinical workflow and obtained proof-of-concept results using fresh tissue specimens from all relevant anatomical sites (nose, temple, eyelid, cheek, forehead, eyebrow and lip). We also investigated the ability to detect the three main types of BCC (nodular, superficial and infiltrative), while varying the analysis times between 20 and 30 minutes.

## 2. Materials and methods

### 2.1 Patient recruitment and tissue collection

Skin tissue specimens (114) were obtained from 112 patients undergoing Mohs surgery at the Nottingham NHS Treatment Centre. Ethical approval was granted by the Health Research Authority (HRA) and Health and Care Research Wales (HCRW) (18/WM/0105) and informed consent was obtained from all recruited patients. Tissue layers were included in the study if their size was smaller than 2 × 2 cm, which is the maximum size that can be measured with the Fast Raman device currently, limited by the size of the tissue cassettes (which have a 2.3 × 2.3 cm fused silica window). If an excised layer was larger than 2 × 2 cm, a sub-section of the layer was cut to fit the cassette, if considered not to intervene with the surgical flow.

### 2.2 Fast Raman measurement

**Fig. 1.**
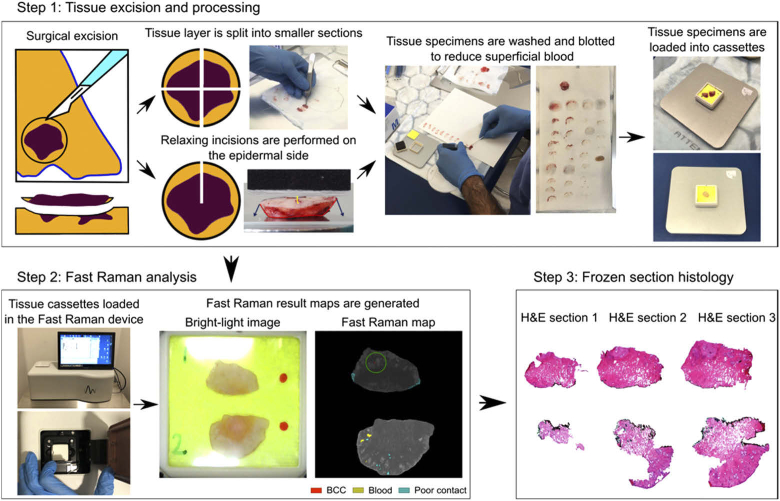
Integration of the Fast Raman device in the pathway of Mohs surgery. Step 1: tissue excision, mapping to maintain orientation, washing and blotting to remove superficial blood. Step 2: Analysis by Fast Raman device – output is presented as an image with BCC presented in red colour and highlighted by a green circle Step 3: Frozen section histology.

The Fast Raman device was integrated into the Mohs departments at the Nottingham NHS Treatment Centre (Nottingham) and Erasmus MC (Rotterdam), where it was used to investigate tissue specimens intra-operatively. The specimens were excised, washed for superficial blood (detailed in the Supplement 1) and loaded in the Fast Raman tissue cassettes (Fig. 1 – Step 1). Cassettes were then loaded into the Fast Raman device, and measurements were started using the instrument controlling software (custom-made software developed in Matlab). The analysis and diagnosis are fully automated. The Fast Raman analysis produced false-colour greyscale maps of the resection surface, where BCC was highlighted as red segments (Fig. 1 – Step 2). To streamline the clinical integration of the Fast Raman device, the analysis time was fixed to 30 minutes. After analysis, the cassettes were removed from the instrument and the specimens were inked and sent to the histopathology lab to be processed for frozen section histopathology, as per standard procedure used in our Mohs units (Fig. 1 – Step 3).

### 2.3 Fast Raman analysis and spectral classification model

To achieve a fixed and predictable measurement time (required to facilitate clinical integration), the acquisition of the Raman spectra was staggered in three rounds, aiming to direct a higher density of sampling points for Raman spectroscopy to regions with a higher BCC probability, while maintaining a fixed total number of spectra. The auto-fluorescence image of the tissue was segmented to screen out collagen rich dermis, producing segments in regions that have a high possibility of being BCC-positive (further described in the Supplement 1). In Round 1, a single Raman spectrum was measured in each of these segments. This round was used to identify adipose tissue and then eliminate these segments from any further Raman analysis. In Round 2, the remaining number of sampling points (up to a maximum of 550) were distributed in the segments classified as non-adipose tissue, maintaining a consistent sampling point density within these segments by using the method proposed by Takamori *et al.* [17]. Segments that had a proportion of BCC detections higher than 45% (empirically optimised) were labelled as BCC positive and the rest of the segments were labelled as BCC negative. In Round 3, 16 spectra were acquired as 4 × 4 raster scans (6 µm step size) in the three segments found to have the highest BCC probability score. The purpose of Round 3 was to screen out false positive segments. Round 3 segments that had more than 75% of the spectra classified as BCC were labelled as BCC-positive, while segments that had less than that were labelled as BCC-negative. If all three segments investigated in Round 3 segments were classified as BCC, all segments labelled as BCC-positive after Round 2 were kept as BCC-positive. If at least one of the three Round 3 segments were labelled as BCC-negative, all Round 2 segments which were labelled as BCC-positive (which therefore had a lower probability of being BCC than the Round 3 segments) were labelled as BCC clear. If none of the Round 3 segments were labelled as BCC positive, the entire tissue sample was declared BCC-clear. After these changes were implemented, the Fast Raman device was able to measure an entire tissue layer up to 2 × 2 cm in 30 minutes. A flowchart of the Fast Raman analysis procedure is included in Fig. S1.

In order to maximise the number of spectra used to train the classification model for each diagnosis, the Fast Raman maps were generated via a leave one patient out cross-validation (LOPO-CV) algorithm. This algorithm was utilised to optimise the parameters and thresholds used by the analysis algorithm, which were selected to produce the Fast Raman maps that best resemble the results of frozen section histopathology (the LOPO-CV algorithm is detailed in Supplement 1). The same set of parameters was used for all Fast Raman maps presented in this study.

## 3. Results and discussions

### 3.1 Raman spectral classification model

**Fig. 2.**
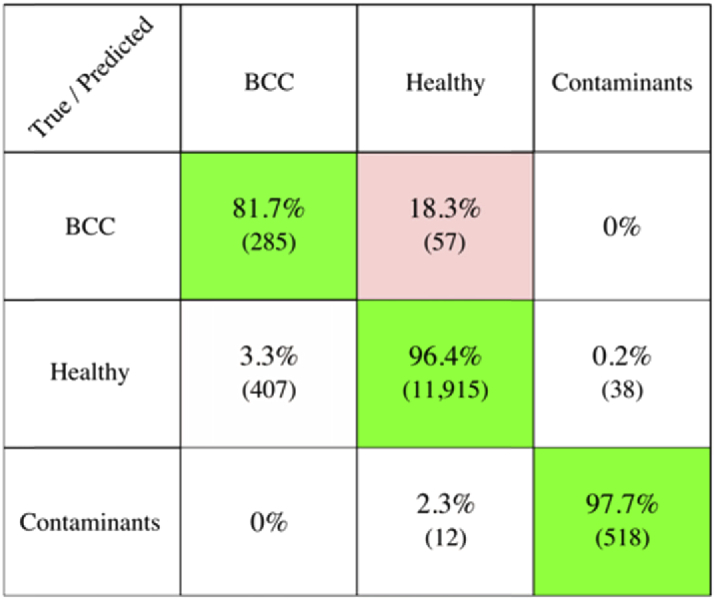
Confusion matrix for the support vector machine (SVM) classification model generated via 5-fold cross validation. The model was trained and validated with spectra acquired intra-operatively during the study. The Healthy group contains spectra of dermis, epidermis, inflamed dermis, fat and muscle. The Contaminants group contains spectra of surgical ink and substrate. Number of spectra for each group presented in parenthesis.

First, we tested the performance of the Raman classification model based on frozen skin specimens reported earlier [18] on fresh skin tissue measured in this study (confusion matrix included in Fig. S2). The results showed a 14% decrease in sensitivity and 2% decrease in specificity when compared to using the same model on frozen tissue specimens. Thus, the spectral classification model was retrained using Raman spectra obtained from fresh skin specimens only (confusion matrix presented in Fig. 2). The 5-fold cross-validation of the support vector machine (SVM) classification model indicated a ∼82% sensitivity and ∼97% specificity when discriminating BCC from all other normal tissue structures. These values are comparable to the performance previously reported for frozen specimens [16–18]. While the re-trained classification model labelled 100% of the non-confounding tissue types (dermis, fat and muscle) as healthy, 37.5% of the spectra belonging to the confounding classes (inflamed dermis, epidermis and hair follicles) were classified as BCC-positive (Fig. S3). However, as the diagnosis of a tissue is not performed on a per spectrum basis, rather on the proportion of BCC detections within a segment, the appropriate selection of decision thresholds from segments in Round 2 and Round 3 compensated for the relatively lower detection accuracy between the BCC class and the confounder classes (∼72%). The full confusion matrix for the fresh tissue classification model is included in Fig. S3.

### 3.2 Evaluation of the surgical excision area analysed by the Fast Raman device

The effectiveness of Mohs surgery depends on its ability to analyse the entire resection surface, aiming to effectively obtain the whole margin onto the slide in the first H&E section. To achieve this, both the deep and lateral margins need to be in one plane for cryo-sectioning. Methods that help this process include the use of relaxing incisions [19], the glass slide embedding technique [20], or cutting 100 µm spaced tissue sections through the specimen [21].

For the Fast Raman analysis, the tissue cassettes were designed to apply gentle pressure onto the tissue block to ensure the tissue is flattened, with both the deep and epidermal margins lying in one plane defined by the cassette window. The Fast Raman device maintains the focus of the laser spot in the plane of the cassette window, ensuring consistent auto-fluorescence and Raman measurements for the entire tissue surface, with a sampling depth of 30 µm. This is consistent with the accepted method for diagnosis in Mohs surgery: a complete section (which includes both deep and peripheral margins) that is negative of tumour can be considered confidently a tumour-clear layer.

Surgical ink and superficial blood were observed to absorb the 405 nm laser light used for auto-fluorescence imaging and to produce high intensity Raman spectral bands, affecting the accuracy of the Raman classification model. To circumvent these issues, tissue inking was delayed until after the Raman analysis and tissue specimens were processed to remove superficial blood (detailed in the Supplement 1). The optimization of the tissue pre-processing (to remove blood) and cassette loading (to increase tissue-cassette window contact) was performed using 83 skin layers from 82 patients. These optimized procedures were then tested using an independent set of 31 layers (30 patients). For this, the instrument software was adapted to automatically calculate and display the percentage of the surface area not analysed by the Raman device due to the presence of superficial blood (yellow) or poor tissue-window contact (blue).

In Table 1, the resection surface area that was not investigated by the Fast Raman measurements is represented as the proportion of the tissue surface that was either out-of-focus or covered by superficial blood. Individual auto-fluorescence generated segments were labelled as blood if more than 70% of the Raman spectra captured within them were detected as blood. Individual segments were labelled as not in contact if all Raman spectra collected within them had a signal to noise ratio below seven (calculated as described in [18]). The percentages reported as not-investigated areas were calculated as the number of pixels covered by either blood or poor contact segments, divided by the total number of pixels that contain the tissue layer within the auto-fluorescence image.

**Table 1.** Percentages of the resection area of tissue specimens analysed by the Fast Raman device (mean/median/standard error).

Tissue type	Nose (n=13)	Eyelid (n=9)	Lip (n=4)	Cheek (n=2)	Forehead (n=1)	Temple (n=1)	Eyebrow (n=1)
Surface area covered by blood (%)	1.35%/0.25%/0.83%	2.21%/1.12%/0.8%	1.59%/1.91%/0.56%	4.78%/4.78%/4.45%	0.02%	1.53%	0.50%
Surface area in poor contact (%)	3.03%/2.27%/0.82%	2.01%/0.98%/0.78%	4.58%/5.10%/1.31%	1.01%/1.01%/0.95%	0.18%	1.42%	2.72%
Total analysed surface area (%)	95.62%	95.78%	93.83%	93.87%	99.81%	97.05%	96.78%

The results show that the fraction of the resection surface area not sampled because of poor contact between the tissue and cassette window ranged between 10.6% (nose section) and 0.18% (forehead section). On average, 2.68% (standard error 0.46%) of the surgical margin was not sampled because of poor contact, with a median of 1.69%. We noted that thicker tissue specimens, which contained predominantly stiffer connective tissue (nose, lip), had a higher proportion of their surface area in poor contact with the cassette window (3.03% and 4.58%, respectively). Tissue sections rich in collagen are known to be challenging to flatten in frozen section histopathology [22]. It has been suggested that such tissue specimens are best flattened against a microscope slide to ensure that as much of the tissue margin is found within a single plane as possible [23]. Glass slide flattening is not standardised across Mohs centres though, and there is no metric to determine how much of the resection surface will be present in the first H&E section, prior to staining. As such, variance still exists when processing tissue, which may lead to sub-optimal diagnoses. For thinner and softer tissue layers (cheeks, foreheads and temples) only ∼1% of the excision surface was in poor contact with the cassette window.

For the 31 test tissue layers, superficial blood covered an average 1.76% (median: 0.36%; standard error: 0.5%) of the total resection area. Tissue specimens which contained more muscle, such as cheeks and eyelids, retained more blood, with an observed coverage of the surface of 4.78% and 2.21%, respectively. On the other hand, skin specimens from forehead and eyebrow had less than 1% area with Raman bands from blood.

While processing of adipose tissue for frozen section histopathology can be challenging due to the lower temperatures needed to freeze such tissue, often resulting in excessive tissue trimming [22], this tissue type poses no difficulties for the Fast Raman measurements. Adipose tissue is easy to flatten against the cassette window and has very low superficial blood.

Overall, the Fast Raman device was able to analyse > 95.56% of the actual resection surface area of the 31 test layers. These results indicate that the tissue cassette applies sufficient pressure to ensure good contact between tissue and the cassette window, and the auto-focusing mechanism of the instrument produces high quality auto-fluorescence images and Raman spectra over the entire surface area. The pre-processing of tissue was also effective at removing the majority of superficial blood. The investigated area could be further increased by performing multiple relaxing incisions on the epidermal side of stiffer specimens to allow better contact between the tissue and the cassette window and by implementing an automated, user-invariant method for washing/blotting of the tissue blocks [19].

### 3.3 Evaluation of skin tissue specimens from various anatomical sites relevant to Mohs surgery

**Fig. 3.**
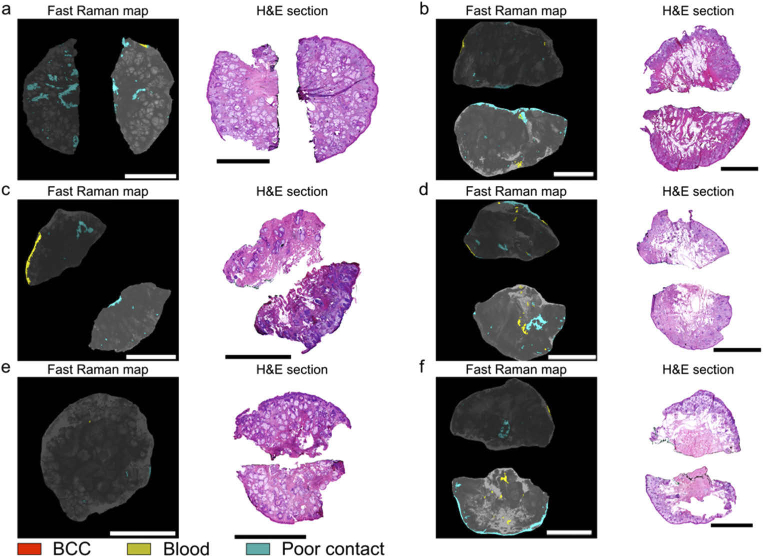
Typical examples of concordant BCC-negative diagnoses by Fast Raman analysis and frozen section histology. Fast Raman analysis of an: a) nose layer, b) cheek layer, c) lip layer, d) eyelid layer, e) forehead layer, f) eyebrow layer. The most relevant frozen sections, showing the majority of the resection surface are included as reference. Scalebars: 5 mm.

After the optimisation of the tissue handling and washing (83 skin layers from 82 patients), 31 tissue layers (31 patients) were used to investigate whether the Fast Raman device is suitable for analysis of skin layers excised at different anatomical locations relevant to Mohs surgery: nose, temple, eyelid, cheek, forehead, eyebrow and lip. Typical examples of Fast Raman measurements are presented in Fig. 3. For all six layers shown in Fig. 3, the diagnosis by frozen section histopathology was BCC-negative, in agreement with the Raman analysis (no red segments present in the Raman images). For these six specimens, the average total investigated surface area was 96.6%, with the highest investigated area observed for the forehead specimen in Fig. 3(e), at 99.8%.

**Fig. 4.**
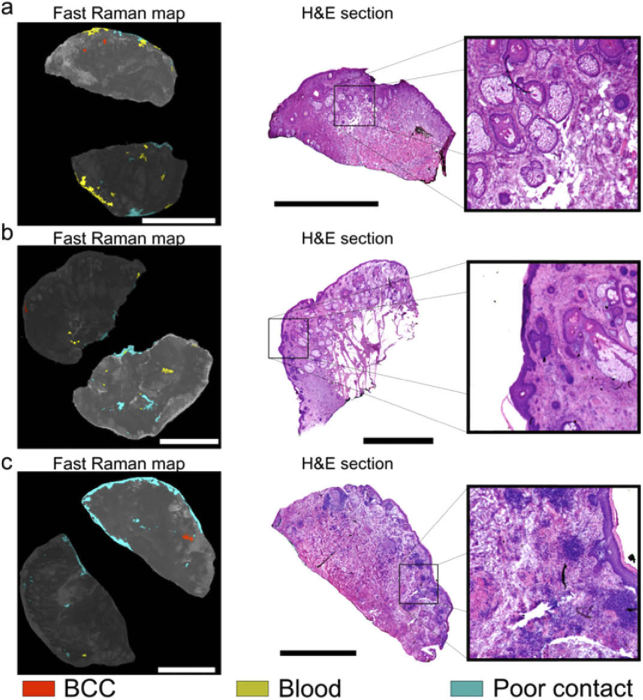
Typical cases of BCC-positive Fast Raman diagnoses declared negative by frozen section histology. Fast Raman analysis of tissue specimens excised from the following anatomical locations: a) lip, b) eyelid, c) nose. The most relevant frozen H&E sections are included. Tissue structures which have been incorrectly detected as BCC and are highlighted in the inset: a) incipient hair follicles; b) incipient hair follicles; c) inflammation. Scalebars: 5 mm.

Considering that the accuracy of the Raman classification model is not 100%, we observed that approximately 10% of tissue layers had segments classified as BCC, which could not be confirmed by frozen section histopathology (Fig. 4). The mean size of the BCC positive segments in these Fast Raman maps was 350 µm in diameter, with a standard error of 70 µm and the largest BCC positive segment was 800 µm in diameter. By comparing the location of the false-positive detections in the Raman maps with the H&E sections, the main confounders for BCC were identified as incipient hair follicles and inflammation. For the tissue layers in Figs. 4(a) and 4(b), the regions predicted as BCC-positive are likely to correspond to incipient hair follicles. While the number of hair follicles in each of the two layers was more than 30 (seen in the frozen sections), only two led to BCC-positive diagnosis. This is more evident for the specimen in Fig. 4(b), for which the H&E section has more than 40 hair follicles and only one BCC-positive detection. For the specimen in Figs. 4(c), the H&E section indicates that the false positive segment corresponds to a region of inflamed dermis. The Fast Raman map only has a single false-positive detection despite the large number of inflamed areas across the tissue surface. While false-positive BCC detections during Fast Raman measurements can still occur, they are usually rare, co-localized and would only result in a small further excision. Even though hair follicles rarely lead to false-positives in frozen section histology, inflammation has been reported to lead to false-positive diagnoses and additional tissue excision in Mohs surgery. In a review of more than 22,000 cases, the presence of inflammation and fibrosis led to excision of additional tissue in 28% of cases. However, histology confirmed the presence of additional tumour only in only 1.9% of these tissue layers [24].

### 3.4 Detection of nodular, superficial and infiltrative BCC

**Fig. 5.**
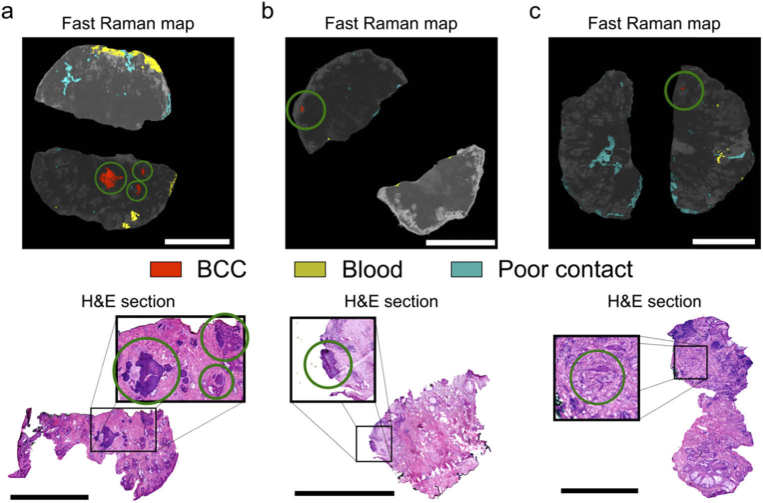
Typical examples of concordant BCC-positive diagnoses by Fast Raman analysis and frozen section histology. Fast Raman analysis of an a) nodular BCC from nose, b) superficial BCC from eyelid, c) infiltrative BCC from nose. The most relevant frozen section, showing the location of the residual tumour for each Fast Raman analysis. Tumours are presented as red segments in the Fast Raman map and are highlighted by green circles. Scalebars: 5 mm.

Next, we tested the ability of the Fast Raman device to detect the three most common types of BCC encountered in Mohs surgery: nodular (Fig. 5(a)), superficial (Fig. 5(b)) and infiltrative (Fig. 5(c)). The smallest single BCC region detected was approximately 100 µm in diameter. Tumours were detected in skin tissue excised from various anatomical locations such as noses, temples, cheeks and eyelids. While only ∼15% of BCCs are infiltrative [25,26], the ability to detect such small BCCs is important as they often penetrate into the deeper layers of skin, making it difficult for surgeons to identify them on the excision margins (Fig. 5(c)).

Furthermore, the Fast Raman device was also shown to detect small residual superficial BCC, such as in the eyelid specimen shown in Fig. 5(b). Cases in which the tumours are located near important organs (such as the eyelid) require very accurate detections, as the surgeons are required to remove the cancerous tissue without affecting tissue functionality. The correct BCC detection and lack of false positive segments ensures that the tumo*u*r would be removed with minimal damage to surrounding tissue for this specimen.

### 3.5 Evaluation and optimizations of fast Raman analysis time

In a previous study using frozen skin specimens, the number of Raman spectra acquired was dependent on the size of the tissue, resulting in a total analysis time that varied between 30 to 70 minutes [18]. As a Mohs surgical excision takes approximately 30 minutes, measurement time was reduced and fixed to 30 minutes to match the schedule during the surgery day, regardless of specimen size.

**Fig. 6.**
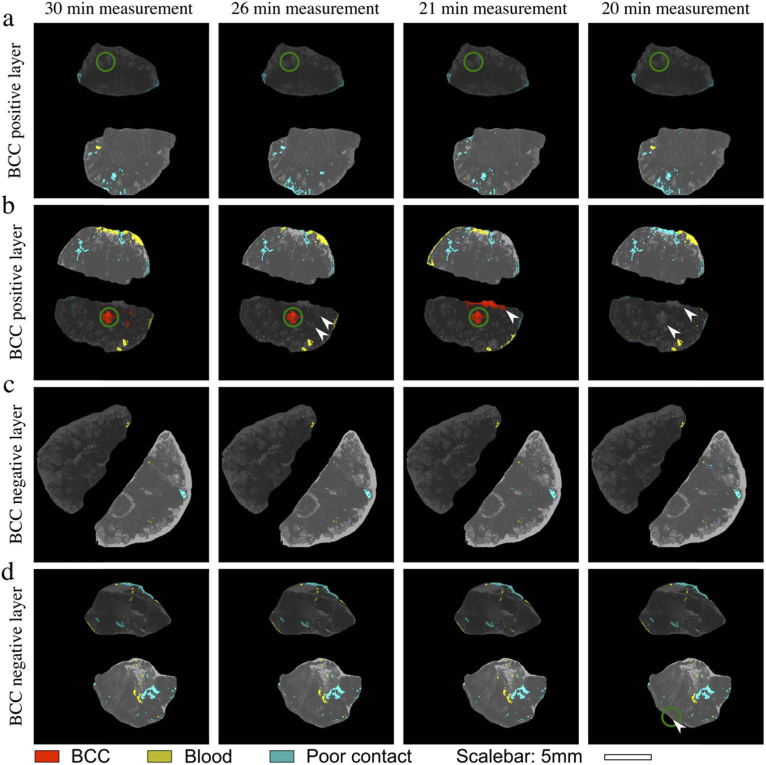
Simulation of reduction of Fast Raman measurement time by addition of noise to Raman spectra. Representative tissue specimens: a) and b) BCC-positive; c) and d) BCC-negative. Measurement time: 30 minutes: 2s/spectrum in all rounds; 26 minutes: 1 s/spectrum in Round 1, 2 s/spectrum in Rounds 2 and 3; 21 minutes: 1 s/spectrum in Round 1 and 2, 2 s/spectrum in Round 3; 20 minutes: 1s/spectrum in all rounds. The locations of tumours are highlighted by green circles. White arrowheads highlight changes in the Fast Raman maps that are caused by the addition of noise. Scalebar: 5 mm.

In order to evaluate the performance of the instrument with an even shorter analysis time, we simulated shorter acquisition times for the Raman spectra by artificially adding Gaussian noise to individual spectra (as reported by other groups [27]). For this part, 77 skin layers from 75 patients were included. Figure 6 presents typical examples of diagnoses obtained as recorded, in 30 minutes (equivalent to acquisition times of 2 s/spectrum in all rounds), and with various simulated analysis times: 26 minutes (1 s/spectrum in Round 1, 2 s/spectrum in Round 2 and 3), 21minutes (1 s/spectrum in Rounds 1 and 2, 2 s/spectrum in Round 3) and 20 minutes (1 s/spectrum in all rounds). Decreasing the acquisition times below 1 s/spectrum led to a significant decrease in performance and it was not evaluated further. The Fast Raman maps of the BCC-positive layer in Fig. 6(a) and the BCC-negative layer in Fig. 6(c) showed no deterioration when noise was added to simulate a measurement time of 20 minutes. In contrast, the BCC-positive layer presented in Fig. 6(b) has become a false-negative detection when the measurement time was reduced to 20 minutes. The BCC-negative layer in Fig. 6(d) shows an incorrect BCC detection when the measurement time is reduced to 20 minutes (highlighted by white arrowhead), but retains its diagnosis when the measurement was reduced to 21 minutes. When noise was added to the Raman signal to simulate the 21 minute acquisition time (1s exposure in Round 1 and Round 2), the diagnosis for all 77 skin layers was the same as for the original 30 minute analysis.

Adjustments to the overhead of the software used to control the device could further reduce the measurement time by ∼2 minutes, resulting in possible <20 minutes measurements per tissue layer. The preliminary results presented here suggest that shortening the analysis time to ∼20 minutes (by reducing the acquisition times of the Raman spectra) would retain the same level of performance as the 30 minute measurements.

## 4. Conclusion

This study reports the first clinical integration of Raman spectroscopy in Mohs surgery as a potential alternative technique for frozen section histology. The Fast Raman device combines auto-fluorescence imaging and Raman spectroscopy in an integrated table-top module that enables analysis of skin tissue specimens without micro-sectioning or staining. Tissue specimens from a wide range of relevant head-and-neck Mohs surgery sites were investigated, and the Fast Raman device was able to correctly detect all sub-types of BCC (nodular, superficial and infiltrative). The analysis can be completed in a fixed and predictable time (30 minutes), which is faster than typical frozen section histology. Our simulations suggest that the measurement time may be further reduced to 20 minutes, without significant changes in detection performance. Completion of the analysis within 20 minutes would allow, if required, for two Mohs layers to be excised while patients are still under local anaesthetic on the operating table. The results show that typically more than 95% of the resection area is analysed by the Fast Raman device, which includes both the epidermal and deep margin, without requiring tissue trimming. Providing “en-face” maps of the tissue is an important advantage compared to histology, where tissue trimming is often required to obtain flat surfaces for sectioning and staining. The Fast Raman device therefore has the potential to provide important benefits to Mohs surgery by reducing the duration and costs of tissue processing and by decreasing diagnosis subjectivity and inter-user variability. However, a larger scale diagnostic test of accuracy study is required to determine the sensitivity and specificity of the Fast Raman device in a clinical setting.

## Data Availability

Data underlying the results presented in this paper are not publicly available at this time but may be obtained from the authors upon reasonable request.

## Supplemental document

See Supplement 1 for supporting content.
