# Supplementary material for: Clinical integration of fast Raman spectroscopy for Mohs micrographic surgery of basal cell carcinoma
Source: Biomed Opt Express. 2021 Mar 11;12(4):2015–26. doi: 10.1364/BOE.417896 (PMC8086475; doi:10.1364/BOE.417896)
Supplement: Supplementary file 1 [file boe-12-4-2015-s001.pdf]

Supplemental Document

Biomedical Optics EXPRESS

Clinical integration of fast Raman spectroscopy for Mohs micrographic surgery of basal cell carcinoma: supplement

RADU BOITOR,[1] COEN DE WOLF,[2] FRANK WEESIE,[2] DUSTIN W. SHIPP,[1] SANDEEP VARMA,[3] DAVID VEITCH,[3] AARON WERNHAM,[3] ALEXEY KOLOYDENKO,[4] GERWIN PUPPELS,[5,6] TAMAR NIJSTEN,[2] HYWEL C. WILLIAMS,[7] PETER CASPERS,[5,6] AND IOAN NOTINGHER[1,*]

[1] *School of Physics and Astronomy, University of Nottingham, University Park, Nottingham, NG7 2RD, United Kingdom*
[2] *Department of Dermatology, Erasmus MC, Rotterdam 3015 GD, The Netherlands*
[3] *Nottingham NHS Treatment Centre, Nottingham University Hospitals, Lister Rd, Nottingham NG7 2FT, United Kingdom*
[4] *Mathematics Department, Royal Holloway University of London, Egham, TW20 OEX, United Kingdom*
[5] *Center for Optical Diagnostics and Therapy, Department of Dermatology, Erasmus MC, Rotterdam 3015 GD, The Netherlands*
[6] *RiverD International B.V., Marconistraat 16, Rotterdam 3029 AK, The Netherlands*
[7] *Centre for Evidence Based Dermatology, Nottingham University Hospital NHS Trust, QMC Campus, Derby Road, Nottingham NG7 2UH, United Kingdom*
[*] *Ioan.Notingher@nottingham.ac.uk*

This supplement published with The Optical Society on 11 March 2021 by The Authors under the terms of the Creative Commons Attribution 4.0 License in the format provided by the authors and unedited. Further distribution of this work must maintain attribution to the author(s) and the published article's title, journal citation, and DOI.

Supplement DOI: https://doi.org/10.6084/m9.figshare.14138273

Parent Article DOI: https://doi.org/10.1364/BOE.417896

Integration of Fast Raman spectroscopy for Mohs micrographic surgery of basal cell carcinoma: supplemental document

Pre-processing of fresh tissue specimens

Tissue specimens were removed according to standard Mohs surgical procedure. Briefly, after local anaesthesia, the central cancer cells were debulked with a curette or scalpel, then the entire area was excised in a circumferential manner with a margin of 1.5-2 mm. The excised layer was divided into 2-4 segments depending on excision size.

After surgical excision, tissue layers were either split into smaller tissue sections or relaxed via incisions to alleviate the tension around the edges of the specimens and facilitate cryo-sectioning for frozen section histology. The tissue specimens were immersed in red blood cell lysis solution (1xRed Blood Cell Lysis Buffer, inVitrogen) for 5-10 seconds, then in saline for 5-10 seconds. The specimens were then delicately blotted and gently squeezed with tissue paper until no traces of blood could be observed on the paper. The tissue specimens were then placed inside tissue cassettes with the resection surface pressed against the cassette window, prior to inking. Marks were made with coloured marker pens on the tissue cassette window to preserve the orientation of the tissue specimens relative to the wound bed.

Overall, tissue pre-processing took 2-5 minutes per tissue layer, depending on tissue type. Tissue specimens that had more superficial blood (e.g. eyelid, cheek) required a more thorough blotting than cleaner tissue specimens (e.g. nose), resulting in slightly longer pre-processing times. After the Fast Raman measurements, the specimens were inked to preserve orientation. This step took approximately 1 minute per layer, though it has to be mentioned that this step is performed as part of the Mohs routine, so no additional time was incurred because due to it.

Frozen section histopathology and Mohs micrographic surgery diagnosis

After the Fast Raman analysis, the cassettes were removed from the instrument and the specimens were inked and sent to the histopathology lab to be processed for frozen section histopathology. Tissue samples were embedded within optimal cutting temperature medium and frozen with cryogenic spray. Samples were then cut with a microtome into 10 μm thick sections alongside the resection surface in 100 μm increments. The sections were then stained with histopathological dyes haematoxylin and eosin. The number of H&E sections that were produced varied for each specimen. Sections were cut from the specimen until the entire epidermis could be observed by the surgeon. Tissue processing for frozen section histopathology takes approximately 45-75 minutes per tissue layer depending on anatomical tissue and experience of Mohs technician.

Using the H&E sections, the surgeons visualise the resection surface by tracking the progression of BCC through the specimen, towards the resection surface. As this is not a physical map, the most representative H&E section (the section closest to the resection surface containing the first occurrence of either tumour or confounding tissue) was displayed throughout the paper, on a per specimen basis.

Segmentation of auto-fluorescence images

The segmentation of auto-fluorescence images was performed by an automated algorithm, which required no user input and was described previously [18].

The first step of the segmentation algorithm was to identify the tissue specimens from the auto-fluorescence image and to separate them from the substrate. To do so, a highly fluorescent cotton pad was placed on top of the tissue specimens, to act as a substrate. An intensity threshold set just below the auto-fluorescence intensity of the substrate was utilised to produce

a mask of each tissue specimen loaded in the cassette. The substrate was removed from the auto-fluorescence image by the mask and the resulting image was flattened with a rotationally symmetric Gaussian low pass filter (to minimize intra-sample intensity variations caused by differences in collagen concentration).

For segmentation, an auto-fluorescence intensity threshold was moved upwards (30 increments) from the lowest auto-fluorescence intensity of the flattened image up to the maximum auto-fluorescence value. For each value of the threshold, all pixels with intensities below it created the segmentation map, resulting in a total of 30 segmentation maps for each layer. Out of the 30 segmentation maps, the optimal segmentation was selected by the algorithm as the one that had both the largest number of segments and the largest total area covered by these segments [18].

As absorption of the 405 nm laser light by blood resulted in an overall decrease in the auto-fluorescence intensity from blood stained tissue specimens (causing large, heterogeneous segments), segments that were larger than 2 mm were further split into smaller segments with the use of a *k*-means clustering algorithm (n = 3 clusters) based on the relative auto-fluorescence intensities within each segment. The smallest segment that can be generated by the auto-fluorescence segmentation algorithm has a diameter of 70 μm and the largest has a diameter of 2 mm.

Leave one patient out cross-validation (LOPO-CV)

To ensure that the estimation of performance is accurate for each iteration of the analysis algorithm during the optimisation process, the accuracy of detection was assessed by leave one patient out cross-validation (LOPO-CV). LOPO-CV utilises data from n-1 patients (where n = 112 patients) to build a classification model which is implemented into the analysis algorithm and used to diagnose specimens from the left-out patient for each iteration. The aim of cross-validation is to test the analysis algorithm's ability to predict data that was not used in its development.

Each spectral classification model was trained n = 112 times, with a different patient being omitted from the training set for each iteration. A target sensitivity of 90% was set for the spectral classification model and the algorithm detection parameters were kept the same for each of the n model iterations.

While LOPO-CV does provide a good approximation of instrument performance, it does have some limitations. As previously mentioned, Raman spectral acquisition was staggered in three rounds, aiming to direct a higher density of sampling points for Raman spectroscopy to regions with a higher BCC probability. The sampling points produced in each round were generated based on model predications from spectra acquired in the preceding round. To reduce the impact of the staggered spectral acquisition on the results and approximate the instrument's performance as accurately as possible, round 3 raster scan measurements were only retained if they corresponded to a BCC-positive segment following the second round of spectral acquisitions. Layers which were diagnosed as BCC positive by Fast Raman, but had no corresponding raster scans to confirm the detection were not retained for the calculation of accuracy, as they do not represent a full Fast Raman measurement.

The algorithm with the highest detection accuracy was selected as the final, optimised analysis algorithm (based on a classification model using SVM with a polynomial kernel of degree 2) and was used to calculate the reported performance estimates.

Figures

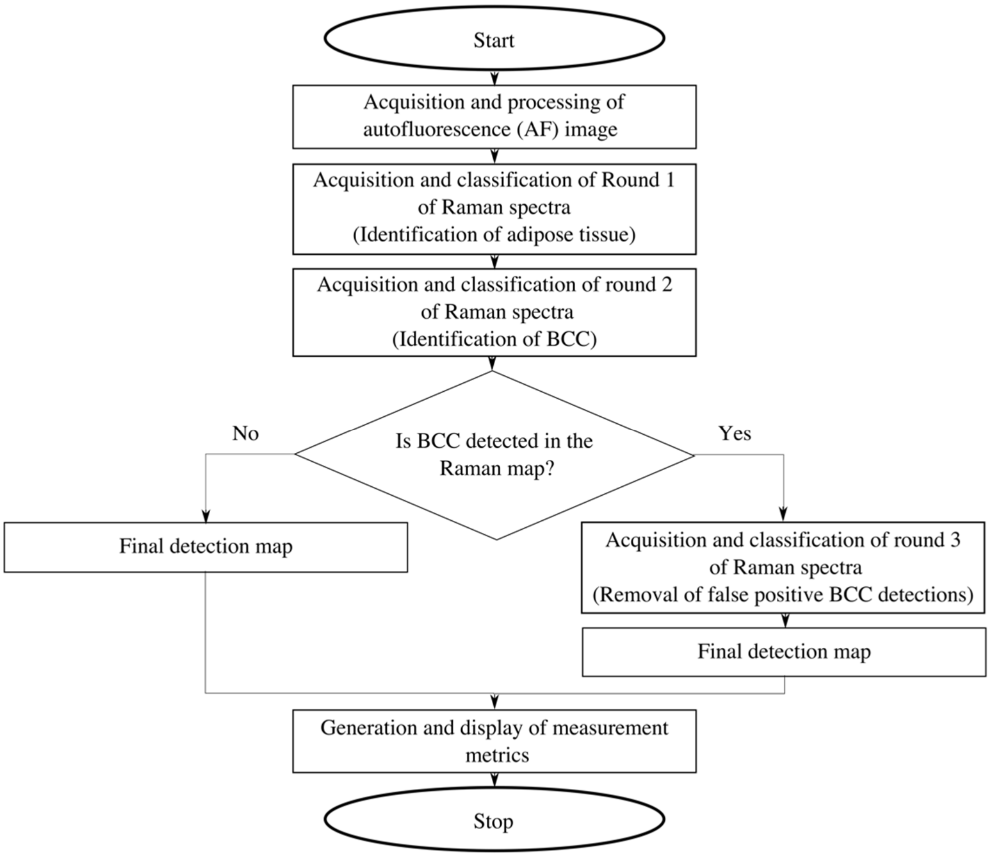

Fig. S1. Flowchart of the Fast Raman analysis procedure.

known\predicted	BCC	Dermis	Blue Dye	Epi + Hair f	Fat	Inflam	Muscle	Red Dye	Substrate
BCC	68.5%	30.7%	0%	0%	0%	0%	0%	0.9%	0%
Dermis	0.3%	91%	0%	0%	0%	0%	0%	8.7%	0%
Blue Dye	12.7%	26.4%	58.9%	0%	0.3%	0%	0%	1.7%	0%
Epi + Hair f	39.9%	47.2%	0%	0%	3.1%	0%	0%	9.8%	0%
Fat	0%	0.5%	0%	0%	99.3%	0%	0%	0.1%	0%
Inflam	13.5%	84.1%	0%	0%	0%	0%	0%	2.4%	0%
Muscle	26.1%	70.8%	0%	0%	0.1%	0%	0%	3%	0%
Red Dye	22.8%	16.3%	53.7%	0%	4.9%	0%	0%	2.4%	0%
Substrate	39.1%	60%	0%	0%	0%	0%	0%	0.9%	0%

Fig. S2. Confusion matrix of the results generated when using the artificial neural network (ANN) classification model, trained on spectra measured from frozen skin tissue, to predict Raman spectra acquired on fresh skin tissue.

known\predicted	BCC	Dermis	Blue Dye	Epi + Hair f	Fat	Inflam	Muscle	Red Dye	Substrate
BCC	81.7%	0%	0%	16.3%	0%	0%	2%	0%	0%
Dermis	0%	99.6%	0.1%	0.2%	0%	0%	0%	0.1%	0%
Blue Dye	0%	0%	95.2%	3.1%	0%	0%	0%	1.7%	0%
Epi + Hair f	45.6%	0.5%	0%	50.8%	0.1%	0%	2.5%	0.5%	0%
Fat	0%	0%	0.1%	0.1%	99.7%	0%	0%	0.1%	0%
Inflam	9.5%	0.4%	0%	11.5%	0%	0%	78.2%	0.4%	0%
Muscle	0.5%	0.1%	0.2%	0.6%	0%	0%	98.1%	0.4%	0%
Red Dye	0%	0%	12.2%	1.6%	0%	0%	0%	84.6%	1.6%
Substrate	0%	0%	2.6%	0%	0.9%	0%	0%	13.9%	82.6%

Fig. S3. Confusion matrix for the support vector machine (SVM) classification model (trained using spectra from fresh skin tissue) based on 5-fold cross validation results on the spectra acquired from fresh skin samples in this study.
